# Nanocrystalline Tin Oxide Nanofibers Deposited by a Novel Focused Electrospinning Method. Application to the Detection of TATP Precursors

**DOI:** 10.3390/s141224231

**Published:** 2014-12-16

**Authors:** José Pedro Santos, Maria Jesús Fernández, José Luis Fontecha, Daniel Matatagui, Isabel Sayago, Maria Carmen Horrillo, Isabel Gracia

**Affiliations:** 1 GRIDSEN, Instituto de Tecnologías Físicas y de la Información (ITEFI-CSIC), Madrid 28006, Spain; E-Mails: mj.fernandez@csic.es (M.J.F.); joseluis.fontecha@csic.es (J.L.F.); daniel.matatagui@ccadet.unam.mx (D.M.); i.sayago@csic.es (I.S.); carmen.horrillo.guemes@csic.es (M.C.H.); 2 Fotónica de Microondas, CCADET, Universidad Nacional Autónoma de México (UNAM), 04510 DF, Mexico; 3 Instituto de Microelectrónica de Barcelona (IMB-CNM-CSIC), Barcelona 08193, Spain; E-Mail: isabel.gracia@imb-cnm.csic.es

**Keywords:** electrospinning, tin oxide, nanofiber sensors, TATP detection

## Abstract

A new method of depositing tin dioxide nanofibers in order to develop chemical sensors is presented. It involves an electrospinning process with in-plane electrostatic focusing over micromechanized substrates. It is a fast and reproducible method. After an annealing process, which can be performed by the substrate heaters, it is observed that the fibers are intertwined forming porous networks that are randomly distributed on the substrate. The fiber diameters oscillate from 100 nm to 200 nm and fiber lengths reach several tens of microns. Each fiber has a polycrystalline structure with multiple nano-grains. The sensors have been tested for the detection of acetone and hydrogen peroxide (precursors of the explosive triacetone triperoxide, TATP) in air in the ppm range. High and fast responses to these gases have been obtained.

## 1. Introduction

One-dimensional nanostructured SnO_2_ with a high surface to volume ratio has attracted special attention in the last years. These types of structures, with different morphologies such as SnO_2_ nanowires, nanobelts, nanorods and nanofibers, are being fabricated via thermal oxidation, thermal evaporation, self-catalytic growth, molten salt synthesis and electrospinning [1–6]. Among the above-mentioned techniques, electrospinning has important advantages such as simplicity, low-cost, and easy mass production. It is one of the most useful methods for the fabrication of 1-D composite nanofibers by electrostatic stretching [6,7].

In the electrospinning process, fibers are usually produced by applying a high voltage to a viscous solution. Under the effect of a strong electric field, a solution drop formed at the end of a metal tip is highly electrified. It experiences two major types of electrostatic forces: a Coulombic force exerted by the external electric field and an electrostatic repulsion between surface charges [8]. With these electrostatic interactions, the droplet is distorted into a conical object (known as Taylor cone). As the voltage reaches a critical value, the electrostatic forces overcome the surface tension of the solution and an electrified jet is produced. The jet is subsequently stretched to form a continuous fiber.

Early works on electrospinning were realized with conventional organic polymers of high molecular weight [9], however as from the middle of this decade, great interest has emerged in the development of metal oxide fibers, such as CuO, SnO_2_, TiO_2_, ZnO, CeO_2_, Ta_2_O_5_, through electrospinning of polymer solutions incorporating metal precursors and annealing processes [10–14]. In the electrospun process various reactions such as hydrolysis, condensation and gelation of the precursors are involved in the morphological and microstructural evolution of the fibers and after a final annealing process at elevated temperatures (≥300 °C), the organic components decompose and the inorganic precursors oxidize forming metal oxide nanofibers on the substrate. Due to their morphological properties, the nanofibers of metal oxides and the coupled oxide nanocomposites nanofibers are being used in gas sensor applications because they improve the gas-sensitive properties of the sensors [15–24]. It is well known that nanostructured shapes such as nanowires and nanofibers are very useful for fabricating gas sensors because of the high conductivity changes produced by the adsorption or desorption of very low concentrations of chemical species on the surface. Therefore in order to use them in gas sensors, it is necessary to identify the morphological parameters that define the surface of the nanofibers. It has been seen by microscopic techniques that individual electrospun nanofibers of oxide materials usually consist of nanosized grains and in addition, nanofiber networks are highly porous, which enhances the process of adsorption-desorption of gases [25,26]. The sensors are often coated with a thin layer of the sensitive material, in which the reaction with the volatiles takes place. In this case the sensitive area equals the active area of the device. The morphology of the sensing layer plays an important role in the molecular adsorption-desorption process, sensor response and sensitivity. In order to take advantage of the larger surface of reaction of the nanostructured materials, they can be used as sensitive layer instead of a thin layer, improving the sensitivity and velocity of the reaction. Therefore, sensitive layers of nanostructured materials could provide better sensitivity than sensitive continuous thin films deposited by methods such as drop, airbrush or spinning.

In the present work, a new method of preparing SnO_2_ nanofibers synthesized by electrospinning is proposed. It involves a focused electrospinning system and annealing process after electrospinning a PVA/SnCl_4_·5H_2_O composite. Scanning electron microscopy (SEM) and X-ray diffractometry (XRD) were used for characterizing the nanofibers. The sensors have been tested for the detection of low concentrations of acetone and hydrogen peroxide, precursors of triacetone triperoxide (TATP) used in improvised explosive devices (IEDs) [27].

## 2. Materials and Methods

### 2.1. Electrospinning Precursor

An electrospinning method was used to synthesize SnO_2_ nanofibers. The electrospun solution was prepared from a PVA/SnCl_4_·5H_2_O composite. First, a solution of poly(vinyl alcohol) (PVA) with molecular weight of Mw ∼170,000 (Sigma-Aldrich, St. Louis, MO, USA) was prepared by dissolving the PVA powder (8%) in deionized water and stirring at 90 °C during 4 h. A solution of tin (IV) chloride pentahydrate (SnCl_4_·5H_2_O, Sigma-Aldrich, 2 g) in deionized H_2_O (2 g) was prepared at room temperature as the precursor material. This solution was slowly added into the PVA solution (20 g) and stirred at room temperature for 2 h. The inorganic/organic composite solution prepared was loaded into syringe with a stainless steel needle whose external diameter was 0.6 mm.

### 2.2. Electrospinning Setup and Electrospinning Process (ESEP)

In the electrospinning process, the solution in the syringe is extruded from the needle tip to the collector, where the device is placed. When high voltage is applied between the needle and the collector, an electrostatic force is induced on the droplets of the solution that are in the needle tip. The interaction between this electrostatic force and surface tension causes the droplets to stretch, forming thin jets of polymer solution which dry in flight and then are deposited onto the collector.

**Figure 1.**
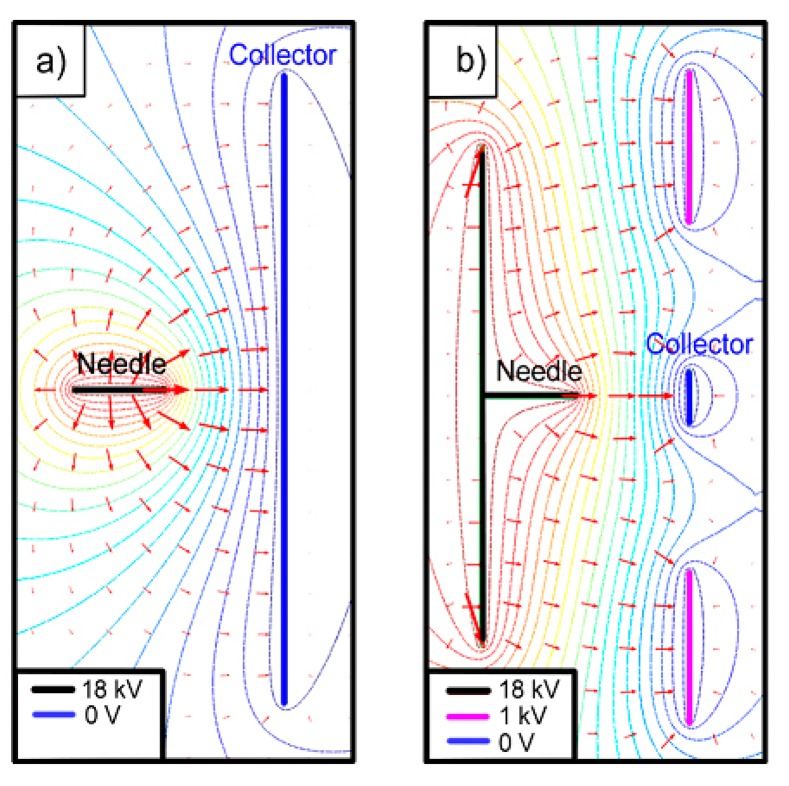
Simulation, using the software Comsol, of the equipotential lines and the direction and intensity of the electric field in the structures described above. (**a**) Usual electrospinning; (**b**) Focused setup used in this work.

In the usual electrospinning setup, the needle is connected to a high-voltage power supply and directed to the grounded collector, a conductor plate, obtaining random fibers, because of the wide distribution of the electric field generated by the needle and the collector when a high voltage is applied between them (Figure 1a). Therefore, it is difficult to electrospin fibers in specific positions. Taking into account that our target area is small (the whole sensor has a diameter of about 10 mm), it is essential to reduce the area of deposition of the nanofibers in order to avoid that they could be torn when the sensor is handled after the nanofiber deposition. Usually the focusing is achieved with electrostatic lens systems, which have ring electrodes between the needle and the substrate [28]. We propose a new simpler method: in plane focusing.

**Figure 2.**
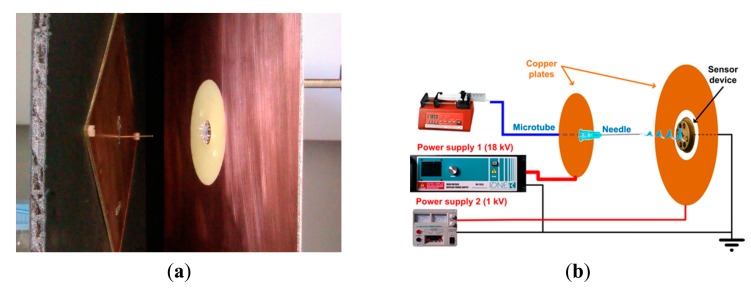
(**a**) Image of the electrodes in the nanofibers deposition: to the left, the source with the needle and the metal screen, both at 18 kV, and in the right part, the focus metal square plate at 1 kV, and the target in its center at 0 V; (**b**) Schematic illustration of the electrospinning setup.

A study of the electric field distribution has been carried out for reducing the fibers deposition area modifying the electrode and collector shape and reducing the needle to collector distance. To perform the required simulations of the modified deposition system, the simulation software Comsol was used. The study took account of the shapes of electrodes, the distance between them and the voltages applied to all them. Figure 1b shows the configuration of the electric field lines when the components are placed in the optimal position and they are fed with the optimal values of the voltages, which finally were used in this work. It can be seen that the electric field lines are concentrated towards the sensor. In fact, we can say that, with this configuration, no fibers were deposited outside the sensor area. A photograph of the nanofiber deposition zone of this structure is presented in Figure 2a. On the left the needle protruding the square metallic screen can be seen and at the right part, the collector structure, consisting of a square metal plate with a central circular window in whose center the sensor is located.

The setup for electrospinning consisted of a 10 mL glass syringe in which a 1 m length microtube was connected in the outlet, which linked the syringe with a metallic needle of 0.6 mm of external diameter. This needle was modified by removing the beveled portion of the tip to let the final cut be perpendicular to its axis. The syringe, was placed in the syringe pump (KD Scientific 210, Holliston, MA, USA) where the solution flow rate was selected, which in this work was optimized to 5 μL·min^−1^. The needle is inserted on a flat 100 mm side square conductor, both fed with a voltage of 18 kV. The collector structure is divided into two parts, an inner circle of diameter 15 mm where the sensor is centered and a 200 mm side metal square whose inner limit is a circle of 60 mm diameter concentric to the circle where the target is placed. The target is set to 0 V and the outer metallic plate is set to 1 kV. The tip of the needle protrudes 15 mm from the square screen and the distance from the needle tip to the sensor is 40 mm. Electrospinning time varied from 60 to 180 s. Figure 2b shows the schematic diagram of the experimental setup.

Nanofibers were electrospun onto two different micromechanized substrates. The first one is a single microhotplate with interdigitated electrodes in a TO-5 package (Cambridge CCS503_17A, Cambridge, UK). The second one is a four microhotplates device in a TO-8 package developed at CNM [29]. Individual sensors could be heated up to 550 °C with power consumption in the order of tens of mW. All substrates with electrospun fibers were annealed between 450 °C and 550 °C in ambient atmosphere for 4 hours using a tube furnace with a temperature ramp of 4 °C/min.

### 2.3. Gas Line Setup

**Figure 3.**
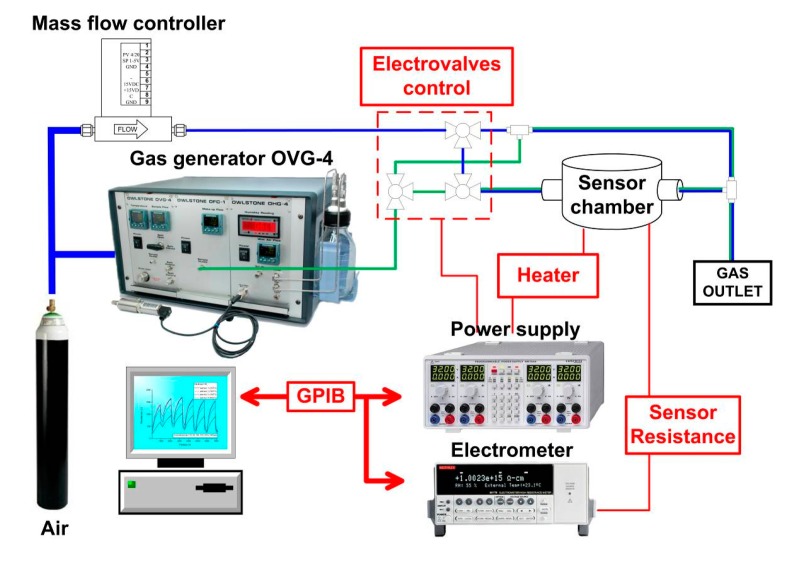
Measurement setup.

A scheme of the gas measurement setup is shown in Figure 3. The vapors were generated by a OVG-4 vapour generator (Owlstone, Cambridge, UK) through calibrated permeation tubes. Sensor resistance was measured by an electrometer with a scanner card (6517 and 6522, Keithley, Cleveland, OH, USA). Voltage for the sensor heaters was provided by a programmable power supply (HM7044, Hameg, Mainhausen, Germany). Both instruments were controlled by a PC through a GPIB interface. Measurements were made at a constant flow of 50 mL/min. Adsorption time was 5 min and desorption times varied between 60 min and 120 min. At least five measurements were made for each concentration and the mean and standard deviation were recorded.

## 3. Results and Discussion

### 3.1. Morphological Characterization

**Figure 4.**
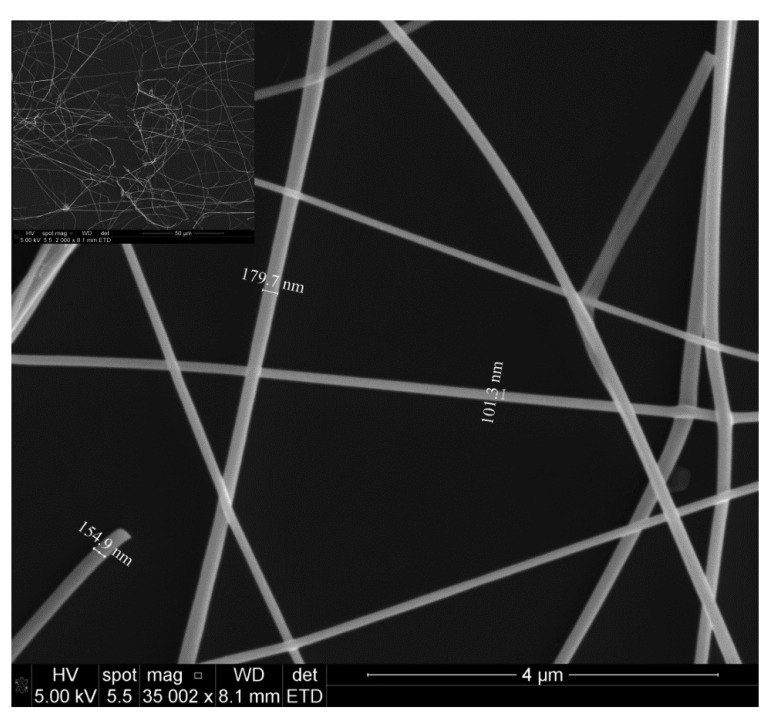
SEM images of SnO_2_ nanofibers annealed at 450 °C for 4 h in air. The inset figure is a low-magnification image, showing the overall feature of the electrospun fibers.

**Figure 5.**
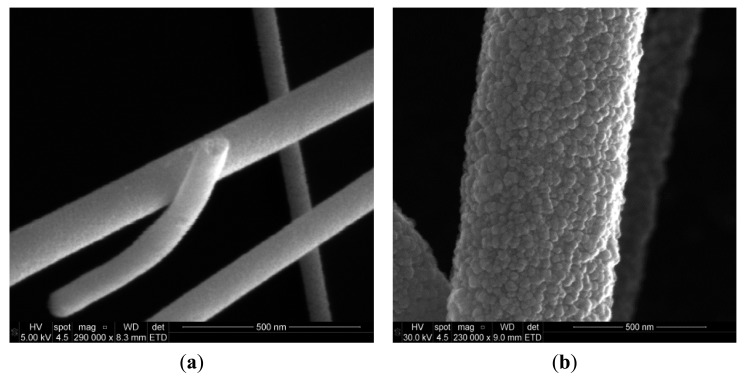
Detail of nanofibers showing their nanostructure. (**a**) Grain sizes of few nanometers after annealing at 450 °C; (**b**) grain sizes of tens of nanometers after annealing at 550 °C.

The microstructure of the fibers was investigated by SEM (Quanta 3d FEG, FEI, Hillsboro, OR, USA) and XRD (D8 Advance, Bruker, Fremont, CA, USA). The SEM images of SnO_2_ nanofibers annealed at 450 °C for 4 h in air are shown in Figure 4. The fibers were intertwined forming porous networks that are randomly distributed on the substrate, as can be seen in Figure 5. In general, the fiber diameters oscillated from 100 nm to 200 nm and fiber lengths reached several tens of microns. It is interesting to note that each fiber had a polycrystalline structure with multiple nanograins as shown in the SEM image of Figure 5 and confirmed by XRD analysis.

The dimensions of these grains increased with annealing temperature and varied from a few nanometers for those annealed at 450 °C (Figure 5a) to tens of nanometers for those annealed at 550 °C (Figure 5b). In next subsection we will see that it will have a great impact in gas sensing properties.

**Figure 6.**
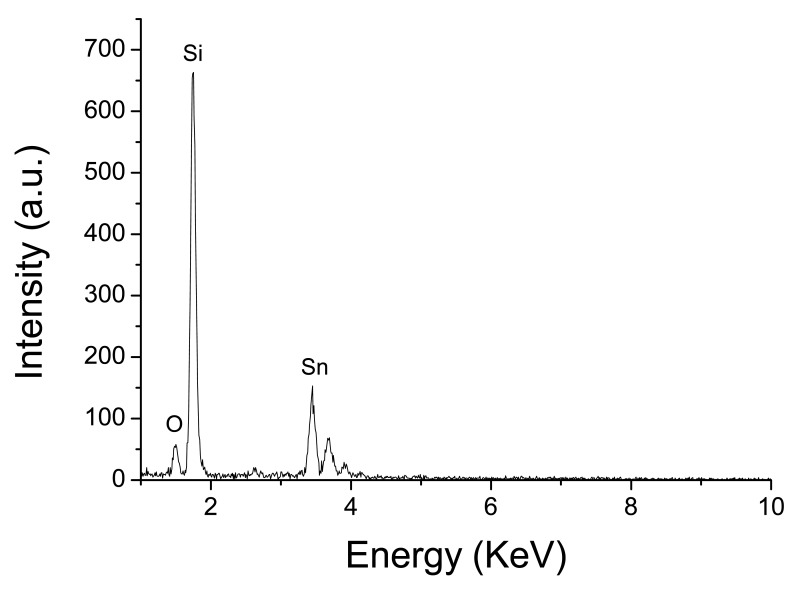
EDX spectrogram of the SnO_2_ nanofiber sensor.

The Energy Dispersive Spectroscopy (EDS) analysis (Figure 6) confirmed that the samples (nanofibers) contain O and Sn atoms. The Si signal was due to the substrate. The X-ray diffractograms show that the fibers are composed by tin dioxide (cassterite) with preferential orientations (111), (211) and (310). From the broadening of the peaks we can estimate the grain sizes applying Scherrer's equation. Results obtained are in good agreement with the values calculated from the SEM images.

The characteristics of the fibers (with multiple nanograins) and porosity of the obtained samples are features particularly useful for gas sensing applications where the surface plays an important role in the detection process [30,31].

### 3.2. Gas Sensing Properties

**Figure 7.**
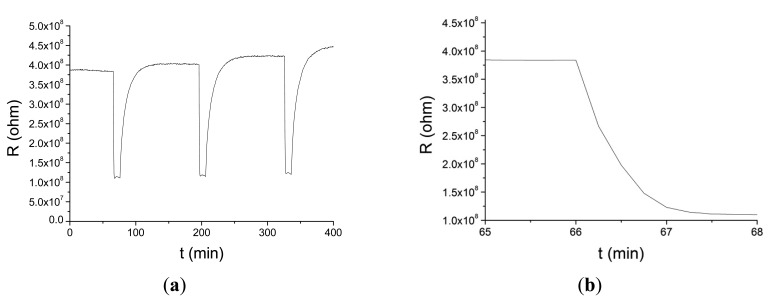
(**a**) Response of a nanofiber sensor with 90 s deposition time and 450 °C annealing temperature to 5 ppm of acetone at 400 °C; (**b**) expanded area around the first adsorption cycle.

These nanofiber-based sensors have been tested in air with acetone at concentrations varying from 2 ppm to 70 ppm and hydrogen peroxide in concentrations from 5 ppm to 180 ppm. The maximum response was obtained around 350 °C–400 °C and with short electrospinning times. In Figure 7a the resistance change of a nanofiber sensor to 5 ppm of acetone at 400 °C is shown. Figure 7b shows a detail of Figure 7a around the first adsorption cycle that shows the fast response time of the sensor.

Sensor temperatures varied from 200 to 400 °C. Resistance of the sensors was in the range from 10^6^ Ω to 10^10^ Ω. Response times were of the order of few seconds (15 s for 2 ppm of acetone at 350 °C) decreasing with operation temperature increase for both compounds. These times are of the same order or lower than those reported for other acetone nanofiber or thin film sensors [32,33].

We have not found any literature reporting hydrogen peroxide nanofiber sensors. The curves are repetitive, although a slight drift is observed. Sensor response for acetone is defined as follows:

(1) $$r(\%)=100\times (R_{a}/R_{s})$$

where *R_a_* is the resistance in air and *R_s_* is the minimum resistance of the sensor exposed to the sample. For hydrogen peroxide the definition is the inverse of Equation (1) because of its oxidizing nature. In the response curves the mean and standard deviations of this value are represented.

**Figure 8.**
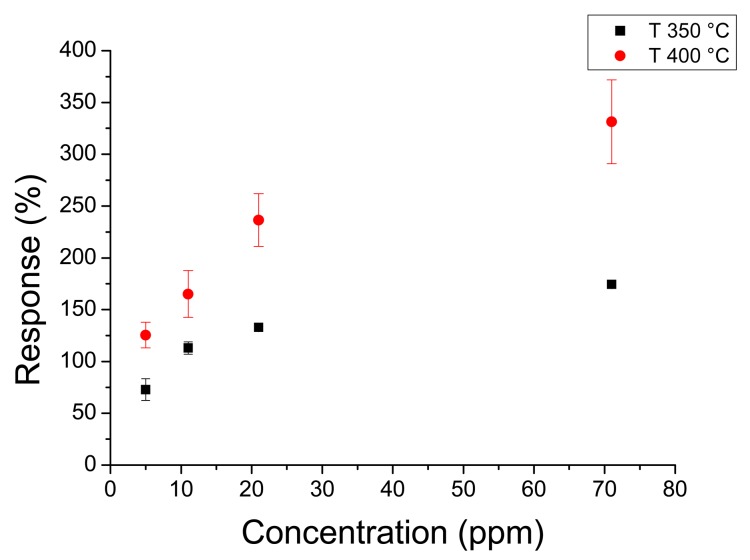
Response of a nanofiber sensor with 120 s deposition time and 450 °C annealing temperature to acetone at 350 °C and 400 °C.

**Figure 9.**
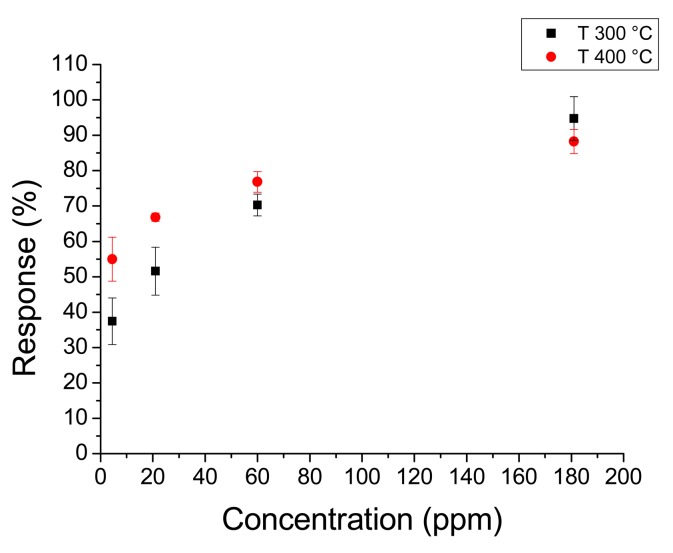
Response of a nanofiber sensor with 180 s deposition time and 450 °C annealing temperature to hydrogen peroxide at 300 °C and 400 °C.

Figure 8 shows the response to the whole acetone range at 350 °C and 400 °C. Figure 9 shows the response to hydrogen peroxide at 300 °C and 400 °C. In this case the response changes with temperature were less remarkable than with acetone. It must be noted that sensors annealed at 450 °C had higher responses than sensors annealed at higher temperatures. In fact sensors annealed at 450 °C had a maximum response higher than 400% to 70 ppm of acetone, whereas sensors annealed at 550 °C did not reach 100% for the same concentration.

For acetone the response mechanism is based on the reaction of the acetone molecule with the adsorbed oxygen:

(2) $${\text{CH}}_{3}{\text{COCH}}_{3}+8{\text{O}}^{-}\to 3{\text{CO}}_{2}+3{\text{H}}_{2}\text{O}+8{\text{e}}^{-}$$

with the releasing of the trapped electrons to the conduction band and decreasing of the resistance [34].

In the case of the hydrogen peroxide the mechanism is the following [35]:

(3) $${\text{H}}_{2}{\text{O}}_{2}+\text{M}\to {2\text{OH}}_{\text{ads}}+\text{M}$$

(4) $$\text{HO}+{\text{e}}^{-}\to {\text{OH}}^{-}$$

where M is a surface adsorption site. The result is the trapping of conduction electrons and thus increasing the resistance.

**Figure 10.**
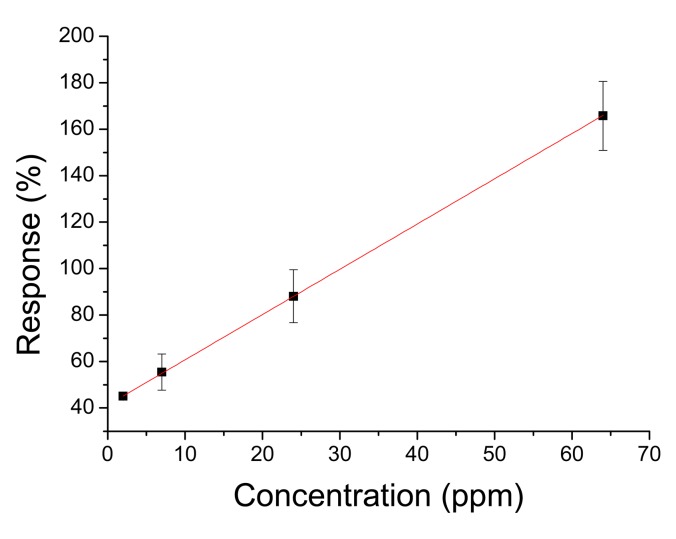
Linear response of a sensor with 90 s deposition time and 450 °C annealing temperature to acetone at 300 °C.

Response times of the order of few seconds are an improvement over thin film sensors that have response times of the order of minutes [35–37]. This is mainly due to the porous structure of the sensors which allows quick gas diffusion, as can be seen from the SEM images. Differences in responses can be attributed to different grain sizes, a well-known effect in nanocrystalline tin dioxide [38–40]. Sensors annealed at 450 °C have smaller grain sizes than sensors annealed at 550 °C, thus the responses of the former are greater than the response of the latter. Although the majority of the responses obeyed to a power law we found a few sensors with linear behavior (Figure 10).

The difference in response curve shapes has been explained in the framework of the electron theory of adsorption where the main parameter is the density of chemisorbed sites at the surface [38]. According to this theory surface oxygen vacancies act as chemisorption sites and small variations from the theoretical value from bulk value lead to different response curve shapes. It has been shown that the tin oxide surface is quite complex and variable oxygen composition of oxide surfaces has been reported [41]. In our case, at same annealing temperature, the only deposition parameter that changes is electrospinning time although it is not clear the way it affects the density of surface adsorption sites and it deserves further research.

## 4. Conclusions

The electrospinning technique is an efficient method for depositing tin oxide nanofibers. We have developed a novel focusing scheme to deposit the fibers onto small areas. The nanofibers were used as sensitive layers for resistive chemical sensors showing high sensitivity, high reproducibility and fast response time in the detection of TATP explosive precursors in the ppm range. The sensors exhibited high and fast responses in a wide operating temperature range. Maximum response values have been obtained with short deposition times at 450 °C annealing temperature. Sensing characteristics as response value and response curve shape were dependent on preparation parameters such as deposition time and annealing temperature.
